# Editorial: Phytoremediation of heavy metal contaminated soil: technology, mechanism, and implementation

**DOI:** 10.3389/fpls.2023.1347564

**Published:** 2024-01-08

**Authors:** Zhaolong Wang, Yongchao Liang, Haibo Liu

**Affiliations:** ^1^ School of Agriculture & Biology, Shanghai Jiao Tong University, Shanghai, China; ^2^ Ministry of Education Key Laboratory of Environment Remediation and Ecological Health, College of Environmental & Resource Sciences, Zhejiang University, Hangzhou, China; ^3^ Department of Plant and Environmental Sciences, Clemson University, Clemson, SC, United States

**Keywords:** phytoremediation, efficiency, mechanism, technology, implementation

Heavy metal contamination has become a severe threat to food safety globally. It is hard to manage the heavy metal-contaminated soil. Heavy metals can easily enter the food chain through cultivated crops, posing a threat to human health (Qin et al., 2021).

Phytoremediation is an environmentally friendly and economical soil remediation technology for heavy metals contamination (Oladoye et al., 2022). The current limitations on the widespread implementation of phytoremediation are mainly due to its low remediation efficiency. It generally takes several decades or even hundreds of years for a successful phytoremediation period (Wang and Delavar, 2023). Improving phytoremediation efficiency requires innovations in both remediation mechanisms and techniques, especially in the discovery and utilization of special plant species with strong capabilities in absorption and accumulation of certain heavy metals, often with the biological mechanism of plant uptake and accumulation of soil heavy metals. The mechanistic aspects of heavy metals accumulation in plants include preferential uptake of heavy metals by roots, their efficient loading into the xylem, translocation from roots to shoots via vascular flux, unloading and accumulation of heavy metals in the shoot tissues, and harvesting and safe disposal of contaminated plant residues after phytoremediation (Muthusaravanan et al., 2018).

In this Research Topic, Sharma et al. reviewed the recent approaches of phytoremediation technologies to remove soil heavy metals, with emphasis on the role of metal-binding proteins in phytoremediation mechanisms, promoting phytoremediation efficiency by adding biochar and flavonoid products, and microbial assistant phytoremediation. Cha et al. revealed a mechanism of nickel (Ni) tolerance enhanced by the bifunctional enzyme YUCCA6 (an auxin biosynthetic enzyme and a thiol-reductase) in Arabidopsis. They found that Ni stress tolerance enhanced by YUCCA6 is contributed mainly by its thiol-reductase activity to reduce the Ni-induced oxidative stress, rather than the elevated IAA levels. Selim et al. found that a newly identified growth-promoting haloarchaeal species significantly reduced cobalt (Co) uptake and mitigated the Co toxicity. In maize, they found that pre-inoculation with haloarchaeal species improved osmoregulation, maintained reactive oxygen species homeostasis, and increased the synthesis of heavy metal-binding ligands (metallothionein, phytochelatins) and the metal-detoxifying enzymes (glutathione S transferase). Zhang et al. reported that an amino acid fertilizer improved the cadmium (Cd) phytoextraction efficiency of *Nasturtium officinal*. They found that the foliar application of amino acid fertilizer not only improved plant growth and antioxidative activity but also promoted root Cd uptake and root-to-shoot translocation. A concentration of 900-fold dilution performed the best with an increase in shoot Cd content by 77%. Zhou et al. compared invasive and native *Phytolaecaceae* plants under Manganese (Mn) stress and the impact on the herbivore (*Spodoptera litura*). They found that hyperaccumulation of Mn in *P. americana* changed the leaf’s secondary metabolites, and inhibited the growth of herbivores by 66%.

The above-cited five papers in this Research Topic have contributed to a better understanding of phytoremediation mechanisms and provided some regulatory pathways to promote the efficiency of phytoremediation technology. Further continued research is certainly required to explore and elucidate new mechanisms of plant uptake and accumulation of soil heavy metals, and innovation of phytoremediation techniques to enhance phytoremediation efficiency because phytoremediation technology applications and practices are long-term complicated processes and hard work. Once efficient phytoremediation technologies are available, widespread implementations of those technologies would be expected for sustainable green development.

## Author contributions

ZW: Writing – original draft, Writing – review & editing. YL: Writing – review & editing. HL: Writing – review & editing.
